# Photo-Stimuli-Responsive CuS Nanomaterials as Cutting-Edge Platform Materials for Antibacterial Applications

**DOI:** 10.3390/pharmaceutics14112343

**Published:** 2022-10-30

**Authors:** Atanu Naskar, Kwang-sun Kim

**Affiliations:** Department of Chemistry and Chemistry Institute for Functional Materials, Pusan National University, Busan 46241, Korea

**Keywords:** photo-stimuli-responsive nanomaterial, CuS, photodynamic therapy, photothermal therapy, antibacterial

## Abstract

Photo-stimuli-responsive therapeutic nanomaterials have gained widespread attention as frontline materials for biomedical applications. The photoactivation strategies are classified as single-modality (based on either reactive oxygen species (ROS)-based photodynamic therapy (PDT), hyperthermia-based photothermal therapy (PTT)), or dual-modality (which combines PDT and PTT). Due to its minimal invasiveness, phototherapy has been extensively applied as an efficient therapeutic platform for many diseases, including skin cancers. However, extensive implementation of phototherapy to address the emergence of multidrug-resistant (MDR) bacterial infections remains challenging. This review focuses on copper sulfide (CuS) nanomaterials as efficient and cost-effective PDT and PTT therapeutic nanomaterials with antibacterial activity. The features and merits of CuS nanomaterials as therapeutics are compared to those of other nanomaterials. Control of the dimensions and morphological complexity of CuS nanomaterials through judicious synthesis is then introduced. Both the in vitro antibacterial activity and the in vivo therapeutic effect of CuS nanomaterials and derivative nanocomposites composed of 2D nanomaterials, polymers, metals, metal oxides, and proteins are described in detail. Finally, the perspective of photo-stimuli-responsive CuS nanomaterials for future clinical antibacterial applications is highlighted. This review illustrates that CuS nanomaterials are highly effective, low-toxic, and environmentally friendly antibacterial agents or platform nanomaterials for combatting MDR bacterial infections.

## 1. Introduction

Bacterial infection has become one of the largest healthcare challenges for humans that requires immediate attention. According to World Health Organization (WHO) predictions, by 2050, ten million people will die every year from infectious bacterial diseases [1]. Moreover, the overuse and misuse of antibiotics are serious issues, as bacteria are becoming increasingly resistant to most available antibiotics [2]. Hence, it is easy to understand the urgent need for a solution or alternative to antibiotics. In this regard, researchers are targeting light sources a modern medical or alternative tool for treating bacterial infections [3]. Different wavelengths of light, ranging from ultraviolet (UV) to near-infrared (NIR), have been utilized with photo-stimuli-responsive nanomaterials to eliminate various bacteria [4]. This method is target-specific and, more importantly, the possibility of bacterial resistance can be avoided.

Reactive oxygen species (ROS)-based photodynamic therapy (PDT) and hyperthermia-based photothermal therapy (PTT) are the two modes of this photoactivated antibacterial activity [4]. Despite the wide use of these approaches for enhancing antibacterial activity, each has its own limitations, such as UV light contamination of healthy tissues in PDT and the high temperature/high dosage of the photothermal agent (PTA) required in PTT [5]. Therefore, researchers have proposed a multimodal strategy in which PDT and PTT can be combined in a single nanomaterial or nanocomposite to achieve more effective antibacterial activity [5]. 

CuS, a p-type semiconductor, is known for its multifunctional properties and is widely used in various biomedical nanomaterials for anticancer, bioimaging, antibacterial, and other applications [6]. The optical properties of CuS are comparable to those of widely used noble metals (Au and Ag), which are extensively utilized for biomedical applications. However, the advantage of CuS over other NIR-absorbing nanomaterials (e.g., Ag and Au) is that the NIR absorption range of CuS is 700–1000 nm. It is also known that the absorption of CuS at >900 nm can be tuned by altering the stoichiometry [6]. Notably, biological tissues have almost no absorption in this NIR region, which in turn enhances the spatial resolution of photoacoustic imaging by reducing background noise and reducing the harm to healthy tissue. 

The photodynamic and photothermal properties of CuS also depend on the morphology of the sample [7], and thus can be controlled by morphology modulation. Moreover, the antibacterial activity can be enhanced by the multimodal strategy of combining PDT and PTT through the use of CuS nanomaterials or CuS nanocomposites [7,8,9,10]. Furthermore, for clinical application in the NIR region, both PDT and PTT can be combined using a singular nanomaterial. 

In this review, we highlight the importance of photo-stimuli-responsive nanomaterials with antibacterial activity and different types of such nanomaterials. The advantages of CuS nanomaterials upon achieving antibacterial action are compared with those of other photo-stimuli-responsive nanomaterials. A detailed description of methods to synthesize dimension-controlled CuS nanomaterials [zero-dimensional (0D), one-dimensional (1D), two-dimensional (2D), and three-dimensional (3D)] is presented, after which the photo-stimuli-responsive antibacterial applications of CuS and its nanocomposites are discussed. Finally, the prospects and challenges of obtaining CuS nanomaterials with antibacterial activity are described.

## 2. Phototherapy and Antibacterial Activity

### 2.1. Importance of Photo-Stimuli-Responsive Nanomaterials with Antibacterial Activity

In recent years, phototherapy has received a great deal of attention for its obvious advantages of minimal invasiveness and clinical safety [11,12]. Moreover, light sources with a specific wavelength range can enable confined irradiation of a pre-defined area using advanced instruments [3]. Niels Ryberg Finsen, the founder of modern phototherapy, showed that the skin disease lupus vulgaris can be treated using sunlight and ultraviolet (UV) irradiation, for which he was awarded the Nobel Prize in 1903 [13,14]. From that point onward, phototherapy has continued to gain increasing importance in biomedical applications. After the introduction of phototherapeutic agents, such as photosensitizers and photothermal conversion agents, the use of phototherapy in many biomedical applications, including anticancer and antibacterial treatments, has grown rapidly [7,8,9,10,15]. Moreover, the development of various phototherapeutic nano-agents has given more impetus to this already important sector [16]. 

In terms of antibacterial activity, phototherapy or photo-activated sterilization has shown immense potential in combatting infectious bacterial diseases and is regarded as an apt alternative to antibiotics that does not induce bacterial resistance [4,5]. In general, photoactivated sterilization can be simplified by utilizing suitable wavelengths of light, ranging from ultraviolet (UV) to NIR, to activate photo-stimuli-responsive nanomaterials. These photo-stimuli-responsive nanomaterials absorb the light energy, inhibiting the growth of pathogens in a short time by producing ROS and/or forming hyperthermic environments [4]. Therefore, this technique can be used to avoid bacterial resistance, which occurs with the long-term use of conventional antibiotics. Moreover, this technique is time-controlled and site specific, i.e., it can selectively target infected sites without damaging other healthy organs or cells. 

### 2.2. Different Types of Photo-Stimuli-Responsive Nanomaterials with Antibacterial Activity

**Figure 1:**
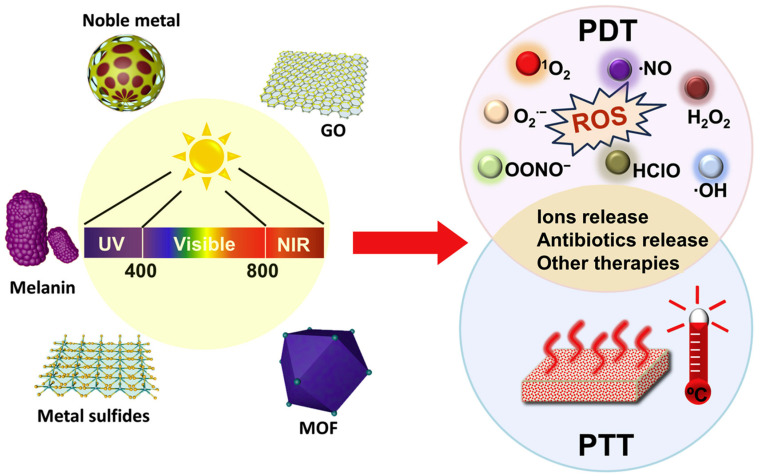
Schematic of photo-stimuli-responsive antibacterial materials and mechanism. Reproduced with permission from Ref. [4]. Copyright 2020 Elsevier.

Photoactivated sterilization (Figure 1), also known as photoactivated antibacterial activity, can be categorized into two types: (1) ROS-based photodynamic therapy (PDT); and (2) hyperthermia-based photothermal therapy (PTT). 

Generally, light of a given wavelength will excite photosensitizers or photocatalysts to their higher excited state, which will then undergo intersystem crossing and generate free radicals and ROS, such as hydrogen peroxide (H_2_O_2_), singlet oxygen (^1^O_2_), and hydroxyl radicals (·OH). ROS are commonly believed to be capable of penetrating bacterial membranes and destroying proteins and lipids, thereby killing bacteria. Therapy based on this light-dependent ROS production and the resulting antibacterial activity is called PDT. ROS can also adhere to the bacterial cell membrane and destroy its defense system [17]. Compared with conventional antibiotics, PDT exhibits significant advantages. PDT enables broad-spectrum therapeutic modes, which can simultaneously act on many metabolic pathways and cellular structures of various bacteria, unlike the singular action mode of antibiotics. Moreover, most photosensitizers used for PDT act only after irradiation with light; therefore, the chance of bacterial resistance is remote. However, most photosensitizers for PDT can be activated only in the UV or visible light range and can be harmful to normal tissue [4,17]. Hence, controlling the clinical range of phototherapy, i.e., NIR-light-activated PDT, might well be the future direction for widespread use. Notably, PDT is less effective against Gram-negative bacteria than Gram-positive bacteria due to differences in the membrane structures [18]. These differences in the ROS activity can be attributed to the dense lipopolysaccharide (LPS) structure outside the cell wall (hard to penetrate) of Gram-negative bacteria and multiple layers of loose, porous peptidoglycan (PG) (easy penetration) in Gram-positive bacteria. This is another limitation of PDT that should be overcome in future studies. Photocatalysts, such as TiO_2_ [19], ZnO [20], WO_3_ [21], and graphitic carbon nitride (g-C_3_N_4_) [22], as well as photosensitizers, such as porphyrins [23], phthalocyanines [24], phenothiazines [25], and up-conversion luminescence nanomaterials [26], have been effectively used in PDT. Au [27], Ag [28], and CuS [4] can also be used to produce surface plasmon resonance (SPR), which can enhance the effect of PDT, also providing effective antibacterial activity. Similarly, carbon quantum dots [29,30] and organic photosensitizers such as indocyanine green (ICG) [31] can be used synergistically for PDT-based antibacterial activity.

PTT is generally regarded as a safer option compared to PDT in terms of its effectiveness and minimal damage to normal tissues [5]. The basic concept of PTT is that under light stimulation, photothermal agents (PTAs), such as noble-metal nanomaterials (e.g., Ag and Au) [32], metallic sulfides (e.g., CuS) [7,8,9,10], carbon-based nanomaterials (e.g., graphene [33] and carbon dots [34]), organic nanomaterials [35], and black phosphorus (BP) [36], can convert solar energy into heat, thereby destroying bacterial cells through hyperthermia. From the earlier days (before the discovery of penicillin), it was recognized that bacteria can be killed at temperatures above 55 °C due to the denaturization of heat-shock proteins [16,37]. Although this approach was previously limited to food preservation, after the invention of PTT, it was regarded as a safe and effective option for killing bacterial cells. NIR light ranging from 700 to 1300 nm is considered as the most non-detrimental light energy for PTT due to its deeper penetration into tissues (more than 3 mm) with minimal damage to healthy tissues, compared to other light sources (e.g., UV and visible), and lower absorption by water and hemoglobin [3]. PTT offers many advantages compared to traditional antibiotic therapy. Firstly, PTT has broad-spectrum antibacterial activity toward various pathogens as the local hyperthermia damages the bacterial structure and disrupts the cell membrane, ultimately inducing bacterial death. Due to this mechanism, the chances of bacteria becoming resistant to PTAs is remote. Furthermore, the treatment time is also short (only a few minutes). Additionally, PTT has the potential to destroy the structure of biofilm by inactivating the inherent bioactive matrix and enabling the penetration of antibacterial agents to kill the protected bacteria [38]. Despite these several advantages of PTT, there are also some limitations that need to be overcome. There is an inevitable chance of damage to normal tissues due to the high power of the excitation light and high dosage of PTAs, which are sometimes needed for hyperthermia treatment [39]. In this regard, the combination of PTT with PDT has recently been considered [7,40]. These multimodal strategies lower the laser density and dosage requirements of PTAs in PTT, and also facilitate cell permeation by ROS generated by the photosensitizers in PDT due to the heat induced by PTT. Therefore, PTT combined with PDT has the ability to not only solve the problems of singular PTT, but also compensate the shortcomings of PDT. Researchers are trying to synthesize nanomaterials that can simultaneously enable PTT and PDT.

### 2.3. Photo-Stimuli-Responsive CuS Nanomaterials with Antibacterial Potential

As discussed earlier (Section 2.2), as isolated strategies, PTT and PDT have intrinsic disadvantages. These include the requirement for high temperatures (PTT), UV-vis irradiation (PDT), and possible damage to healthy biological tissues [5]. Therefore, researchers are trying to design a novel synergistic antibacterial platform, where the advantageous properties of both PDT and PTT are combined and the limitations of each therapy are compensated. CuS is a potentially superior nanomaterial, as it shows both PTT and PDT under irradiation with different wavelengths of light [7,8,9,10]. 

CuS, a semiconducting metal chalcogenide with a narrow bandgap, displays suitable photocatalytic and exceptional photothermal properties [7]. Most importantly, CuS is considered as an excellent PTA due to its excellent potential for antibacterial activity. The light-to-heat conversion characteristics of CuS nanomaterials can be attributed to the d–d energy band transition of Cu^2+^ ions [41]. Due to the excellent NIR absorption and conversion ability of CuS, CuS nanomaterials have the potential to effectively eradicate pathogenic infection. Cu^2+^ is also considered one of the most important trace elements in the human body, playing an essential role in stimulating the wound healing process [42]. The basic advantage of CuS, along with other widely used photoactive nanomaterials, such as Ag and Au, is the ability to absorb NIR light by means of a localized surface plasmonic resonance (LSPR) originating from the collective oscillation of holes in these materials. However, for noble metals, the electrons act as carriers for the same LSPR phenomenon [43]. Unlike noble metals, the LSPR position of CuS can be controlled by modulation of the number of carriers. Hence, relative to other samples, this feature has given CuS-based nanomaterials a significant push toward photoactive applications (e.g., anticancer and antibacterial), especially under NIR irradiation. 

Furthermore, CuS exhibits excellent antibacterial properties when doped with metals or non-metals. For example, a CuS/protonated g-C_3_N_4_ composite showed effective antibacterial activity against Gram-positive and Gram-negative bacteria due to collaborative photodynamic and photothermal bactericidal effects [44]. Similarly, Mutalik et al. [7] demonstrated the potential of CuS with morphology-dependent photodynamic and photothermal antibacterial activity against *E. coli*, facilitated by NIR light. Zhou et al. [45] presented a CuS hybrid hydrogel with the potential to completely eradicate *E. coli* and *S. aureus* via photothermal/photodynamic effects under NIR laser irradiation. A collection of poly(5-(2-ethyl acrylate)-4-methylthiazole-*g*-butyl)/copper sulfide nanoclusters (PATA-C_4_@CuS) was also synthesized to effectively kill levofloxacin-resistant Gram-negative and Gram-positive bacteria after NIR laser irradiation [9]. The dual function of PDT and PTT was exploited to achieve effective antibacterial activity. Qiao et al. [46] also demonstrated similar antibacterial potential of CuS nanodots against methicillin-resistant *S. aureus* (MRSA) and extended-spectrum β-lactamase *E. coli* by combining PTT and PDT with NIR laser irradiation. More recently, our group [47] published a BP-based CuS nanoplatform with similar antibacterial activity against *P. aeruginosa* and *S. aureus* in response to NIR light, along with ROS generation.

Despite the numerous advantages of CuS nanomaterials for multimodal PDT and PTT, there are still some limitations to overcome before these materials can be finally utilized for some worthy clinical applications. The toxicity of CuS remains an issue requiring immediate resolution; toxicity is an ever-present issue for nanomaterials for clinical applications. Additionally, the synthesis of CuS is slightly more complex than that of certain other nanomaterials. Therefore, a simple and cost-effective route needs to be established for the synthesis of CuS to facilitate related antibacterial applications. In addition, the required dosage of photo-stimuli-responsive nanomaterials (such as CuS) is still higher than that of antibiotics, which requires more attention when used in clinics. The nondegradable property of photo-stimuli-responsive CuS nanomaterials, along with the phototoxicity of light, are areas that require significant future emphasis.

## 3. Controllable Synthesis of CuS Nanomaterials

Nanomaterials have attracted great attention for biomedical applications due to their unique physical, chemical, and electronic properties [48,49], which are significantly dependent on the dimensions of the nanomaterials, making them important entities for various applications. We classified the synthesis of CuS nanomaterials in terms of the dimensions (zero-dimensional (0D), one-dimensional (1D), two-dimensional (2D), and three-dimensional (3D)). Various synthesis techniques have been utilized to synthesize CuS nanomaterials with different dimensions, as the morphology of the CuS nanomaterials may vary with the synthesis processes, eventually affecting the final applications. For example, spherical CuS nanomaterials [50] are mainly used in photoacoustic imaging and therapeutics, along with photothermal ablation, whereas hollow nanocubes and nanocages are utilized in drug delivery [51,52]. Furthermore, CuS nanorods and nanowires have been successfully used in biosensing applications to detect food pathogens and immunologically relevant moieties [6]. Therefore, it is easy to agree on the fact that the synthesis technique is a determining factor for CuS nanomaterials and on the corresponding biomedical applications. Therefore, the synthesis of CuS nanomaterials, such as CuS nanoparticles (0D), CuS nanorods, nanowires, nanotubes (1D), CuS nanoplates (2D) and nanospheres, nanocages, and nanoflowers (3D), are discussed according to the dimensionality of the samples.

### 3.1. Synthesis of Zero-Dimensional (0D) CuS Nanomaterials

Despite the availability of various methods of synthesizing 0D materials, only a few 0D CuS nanomaterials have been successfully prepared. Qiao et al. [46] successfully synthesized CuS nanodots by a facile single-step hydrothermal route, where a biocompatible and highly soluble protein, bovine serum albumin (BSA), was utilized to control the particle size and stability of the sample. The CuS nanodots not only eradicated multidrug-resistant bacteria, but also expedited wound healing due to their NIR light-mediated photothermal effect. In another experiment, CuS quantum dots (QDs) were obtained via green synthesis [53]. A transparent solution of starch, which is a natural biodegradable polymer, was used as both a reducing and capping agent for the synthesis of CuS QDs. Moreover, the size of the synthesized CuS QDs was controlled by adjusting the reaction parameters. The CuS QDs selectively sensed Hg^2+^ ions, demonstrating potential toward sustainable nanotechnology. These syntheses demonstrate that 0D CuS nanomaterials can be obtained by simple approaches, and we envision numerous potential biomedical applications of 0D CuS nanomaterials in the future.

### 3.2. Synthesis of One-Dimensional (1D) CuS Nanomaterials

The excellent optical, magnetic, and mechanical properties of 1D nanomaterials are always attractive for researchers [54]. Due to their enhanced catalytic and electrochemical characteristics, 1D CuS nanomaterials are mainly used for biosensor application [6]. However, attempts have been made to use them for other biomedical applications, especially as antibacterial agents [55]. Notably, 1D CuS nanomaterials can vary in diameter from 1 to 100 nm, with nanotube, nanorod, and nanowire morphologies [54].

A wide range of approaches for synthesizing 1D CuS nanomaterials have been explored, varying from hydrothermal to template-assisted, high-pressure autoclave, and other approaches [6]. Along with the synthesis process, the composition, growth conditions, and precursors also play important roles in the formation of 1D CuS nanomaterials, such as CuS nanotubes (30–120 nm) and nanowires (30–80 nm). The basic advantage of CuS nanorods/nanotubes for biomedical applications is their intrinsically high surface area. Bekhit et al. [55], synthesized CuS nanotubes via a green, simple, and effective gamma-radiolysis method without adding any capping or reducing agents. The synthesized CuS nanotubes showed broad-spectrum antimicrobial activity against *Aspergillus niger, Bacillus subtilis*, *Escherichia coli*, *Pseudomonas aeruginosa*, *Staphylococcus aureus*, and yeast. CuS nanotubes have also been utilized in various biomedical applications, including biosensor and anticancer applications [56]. Similarly, CuS nanorods have been synthesized via various methods, such as microwave irradiation and hydrothermal techniques. Manikandan et al. [57] utilized a hydrothermal method to synthesize cellulose acetate (CA)-templated nanorods like CuS fibers using vegetable extract (*Brassica oleracea* var. *italica*). This vegetable extract itself acts as a reducing agent. The synthesized CuS nanorods showed effective antibacterial activity against *E. coli* and *Bacillus cereus*. CuS nanowires are another nanomaterial that has captured research interest due to their basic advantage of a high contact area [58].

### 3.3. Synthesis of Two-Dimensional (2D) CuS Nanomaterials

Although 1D and 3D CuS nanomaterials have been widely synthesized for many applications, including antibacterial agents, 2D CuS nanomaterials are comparatively less explored and limited to nanoplates and thin films [59,60]. Because the biomedical applications of these nanomaterials are not extensively reported, we herein only briefly discuss the structures and related syntheses. 

Similar to 1D CuS, hydrothermal and solvothermal approaches are the most explored approaches for synthesizing 2D CuS nanomaterials, where the size of the plates range from 50 to 200 nm. To exploit their antibacterial activity, Mirzaei et al. [59] synthesized CuS nanoplates and anchored them onto g-C_3_N_4_ nanosheets suspended in a poly(methyl methacrylate) (PMMA) matrix. A hydrothermal method was also used to synthesize CuS nanoplates and the antibacterial activity against *E. coli* and *S. aureus* was assessed, confirming the antibacterial properties of the CuS nanoplates. Nishi et al. [60] successfully synthesized CuS nanoplates for potential localized surface plasmon resonance (LSPR)-mediated biosensors. Sonochemical [61] and chemical vapor methods were also used to synthesize CuS nanoplates [62].

### 3.4. Synthesis of Three-Dimensional (3D) CuS Nanomaterials

Researchers have always preferred 3D nanomaterials in comparison with their 0D, 1D, and 2D counterparts for various applications, including biomedical applications. This is due to the unique high surface area and large surface-to-volume ratio, along with the greater structural stability of 3D nanomaterials [54]. Hence, it is easy to understand the extensive use of 3D CuS nanomaterials, such as CuS nanospheres [63], CuS nanocages [64], hierarchical CuS nanomaterials [65], CuS microflowers [66], and hollow CuS [51], for various applications compared to the 0D, 1D, and 2D versions. Several methods, such as the sol–gel, hydrothermal, solvothermal, microwave, and sonochemical approaches, have been used to synthesize 3D CuS nanomaterials [6]. 

The hydrothermal method seems to be the preferred choice for researchers due to the ready availability of water, low cost, and environment friendly nature. Water can dissolve non-ionic covalent compounds with the use of high temperature and pressure. Water can also be used as both a pressure-transmitting medium and solvent medium, which in-turn lowers the activation energy of the reactions. For example, sodium thiosulfate (Na_2_S_2_O_3_) and copper sulfate (CuSO_4_) aqueous solutions were mixed in deionized water and transferred into Teflon-lined stainless-steel autoclaves for hydrothermal reaction at 180 °C for 12 h to obtain CuS nanospheres [67]. Shu et al. [68] utilized a facile one-pot, template-assisted hydrothermal method to synthesize porous hollow CuS nanospheres. AL-Jawad et al. [69] utilized the hydrothermal process to prepare CuS nanomaterials with strong antibacterial potential against *E. coli*, *P. aeruginosa*, and *S. aureus*. 

Microwave irradiation is another simple and widely used approach for synthesizing CuS nanomaterials which is also fast and energy efficient. For example [70], microwave irradiation of a mixture of Cu and S precursors in an aqueous medium yielded CuS nanomaterials in a specific timeframe. 

Han et al. [63] utilized a simple template-assisted approach to synthesize 3D hollow CuS nanospheres, which were further used for the controlled delivery of doxorubicin, improving the photothermal-chemotherapeutic effect. 

From the above discussion of the synthesis methods, it is clear that various factors, such as the precursor salt, solvent, reaction temperature, applied pressure, and surfactant, govern the synthesis of various CuS nanomaterials, which in turn directs the resulting applications. 

## 4. Photo-Stimuli-Responsive Antibacterial Applications of CuS and Its Nanocomposites

As discussed in Section 2.3, CuS has excellent photoactivated antibacterial properties and shows promising antibacterial activity when irradiated in the so-called biological window (i.e., NIR region) [71]. In this section, we further discuss the photo-stimuli-responsive antibacterial applications of CuS and its nanocomposites (Table 1).

### 4.1. CuS Nanomaterials

**Figure 2:**
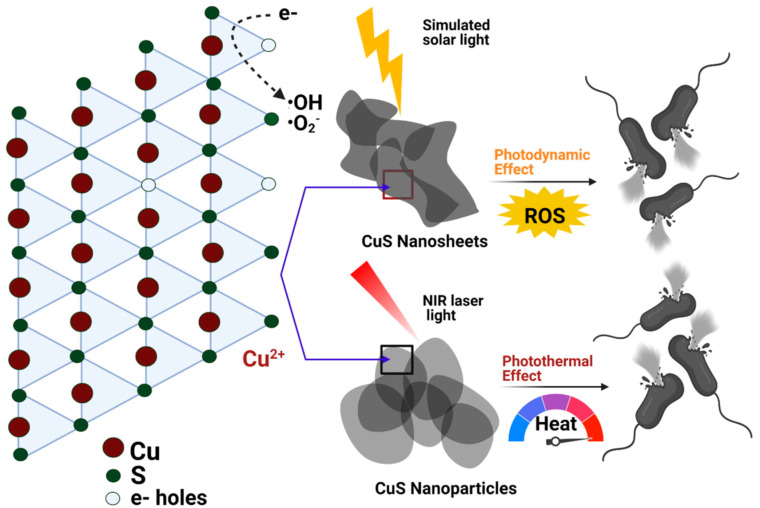
Schematic illustration of CuS NSs and NPs mediated photodynamic and photothermal antibacterial pathways. Reproduced with permission from Ref. [7]. Copyright 2022 Elsevier.

CuS nanomaterials exhibit inherent photoactivated antibacterial potential, especially in the NIR region. For example, Qiao et al. [46], developed ultrasmall copper sulfide (covellite) nanodots (CuS NDs), which showed effectiveness in the treatment of multidrug-resistant bacteria-infected chronic non-healing wounds. The dual functional mode of the CuS nanosystem enabled eradication of extended-spectrum β-lactamase (ESBL) *E. coli* and methicillin-resistant *S. aureus* (MRSA) in both in vitro and in vivo studies, while simultaneously expediting wound healing. The NIR laser (808 nm, 2.5 W·cm^−2^)-mediated photothermal effect and remote control of copper-ion (Cu^2+^) release were the main origins of the dual-mode activity. Ren et al. [72] utilized CuS nanoparticles (NPs) to prepare a photothermal antibacterial silk fabric. A chitosan quaternary ammonium salt (QCS) was used as a template. The antibacterial efficiency of CuS NP-deposited silk fabric with QCS as a template (SF/QCS/CuS) resulted in 99.99% bacterial death of *S. aureus* and *E. coli* within 5 min of irradiation (400 mW·cm^−2^). The nanocomposite also showed good UV-resistance and superb light-to-heat conversion efficiency. Even after 10 washes, the antibacterial activity of the nanocomposite remained at a high level, demonstrating excellent bonding between the photothermal nanomaterials. In a similar experiment, CuS NPs were coated onto glass slides, and the antibacterial properties were investigated [43]. The CuS NP–glass samples showed good stability and an admirable photothermal effect when irradiated with NIR light. The samples were also effective for the controlled release of copper in water. Hence, the antibacterial activity of the CuS NP-glass samples against *E. coli* and *S. aureus* was attributed to the dual mechanism of hyperthermia-induced NIR laser irradiation and the slow and sustained copper release. As discussed earlier, CuS has shown morphology-dependent photodynamic and photothermal antibacterial activities [7]. This study also proves that CuS itself can be employed for simultaneous ROS-mediated photodynamic therapy and hyperthermia-mediated photothermal therapy (Figure 2). A hydrothermal process was utilized to synthesize CuS microspheres (MSs), CuS nanosheets (NSs), and CuS nanoparticles (NPs) by using three different solvents (ethanol, glycerol, and water, respectively). In this case, the antibacterial activity against *E. coli* was attributed to ROS production and heat generation after irradiation with simulated solar and NIR light. Thus, all the above reports on CuS demonstrate its immense inherent photoactive antibacterial potential. However, CuS can also be utilized in nanocomposites by combining it with a polymer, 2D nanomaterial, metal, protein, or other material, as discussed in the ensuing sections.

### 4.2. CuS with 2D Nanomaterials

In recent times, 2D nanomaterials have revolutionized research due to their unique physiochemical properties, non-toxicity, and excellent utility in various biomedical applications, including antibacterial treatments [79,80,81]. After the immense success of graphene [82,83], researchers have been exploring different 2D nanomaterials as antibacterial agents, namely graphitic carbon nitride (g-C_3_N_4_) nanosheets [22,59], titanium carbide (Mxene) [84], boron nitride (BN) [85], transition metal dichalcogenides [e.g., molybdenum disulfide (MoS_2_)] [86], BP [36,47,87,88], and others. The basic advantage of using 2D nanomaterials with CuS is the synergetic improvement in the antibacterial activity, with less chance of toxicity, due to the biocompatibility of 2D materials.

**Figure 3:**
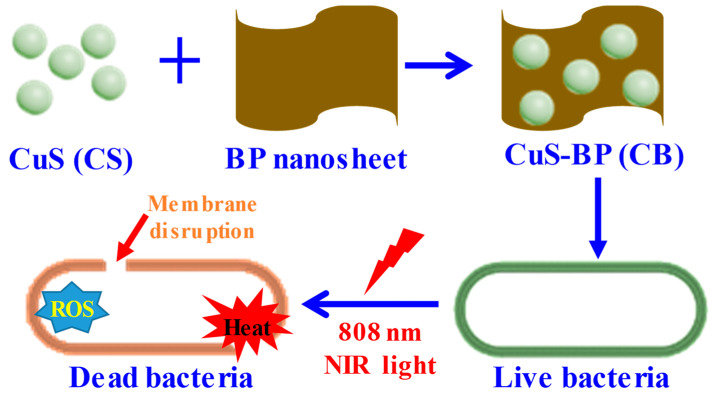
Schematic of CuS-BP synthesis and its PTT- and PDT-dependent antibacterial activity. Reproduced with permission from Ref. [47]. Copyright 2022 Elsevier.

In this respect, Lv et al. [73] demonstrated the importance of CuS nanocomposites with graphene oxide (GO) for photothermal antibacterial applications by utilizing visible light. A simple one-pot hydrothermal method was utilized to synthesize flower-like CuS anchored on GO sheets. Due to the morphology-dependent high surface area of CuS and defects in GO, the nanocomposite showed excellent photocatalytic performance. The nanocomposite showed excellent antibacterial activity against *E. coli* and *S. aureus*. This antibacterial activity was attributed to the excellent photocatalytic activity of the nanocomposite, coupled with the visible-light-induced (0.2 W·cm^−2^) photothermal response and release of Cu ions. The nanomaterial was also biocompatible with L929, a murine fibroblast cell line. Another 2D nanomaterial, graphitic carbon nitride (g-C_3_N_4_) nanosheets [74], were utilized with CuS nanomaterials for the photothermal eradication of bacteria. In that study, a simple hydrothermal process was again utilized to fabricate CuS nanomaterial-decorated graphitic carbon nitride (g-C_3_N_4_) nanosheets. Thereafter, polyethylene glycol (PEG) was capped with the nanocomposite to make it more biocompatible. The PEG-CuS@g-C_3_N_4_ nanocomposite was effective as an antibacterial agent, killing up to 99% of both *E. coli*. and *S. aureus* in a 200 µg·mL^−1^ suspension within 20 min of NIR irradiation (808 nm, 2.5 W). The biocompatibility of the nanocomposite was also verified using mouse skin fibroblast NIH-3T3 cell lines. Ti_3_C_2_T_x_ Mxene was also used with CuS to achieve PDT- and PTT-mediated multimodal activity against *E. coli* and *S. aureus*, with bactericidal rates of 99.6 and 99.1%, respectively [8]. Ti_3_C_2_T_x_@CuS composites were synthesized by a simple hydrothermal method, where the CuS NPs were distributed on the surface of Ti_3_C_2_T_x_, affording synergistic effects. ROS generation, which eventually facilitated destruction of the bacterial cells, was attributed to the formation of the Ti_3_C_2_T_x_@CuS heterojunction, which promoted the separation of electrons and holes, leading to improved electron transport efficiency. Furthermore, the nanocomposite had a stronger photothermal effect than the individual components, confirming its synergistic action. Recently, our group reported a BP-based CuS nanoplatform (CB) with photothermal activity, demonstrating antibacterial activity against environmental bacterial pathogens [47]. In our work, a low-temperature solution synthesis method was utilized to prepare CuS nanomaterials, which were then immobilized on BP nanosheets. The synthesized CB nanocomposites showed excellent antibacterial activity against *P. aeruginosa* and *S. aureus*, and also showed good activity against the MDR strains of these bacteria after irradiation with NIR light (808 nm, 2.5 W). The CB nanocomposite also showed exceptional ROS generation ability under NIR irradiation relative to the non-NIR-irradiated sample. Therefore, the multi-modal action of ROS and hyperthermia was also successfully applied therein under NIR irradiation (Figure 3).

### 4.3. CuS with Polymers

**Figure 4:**
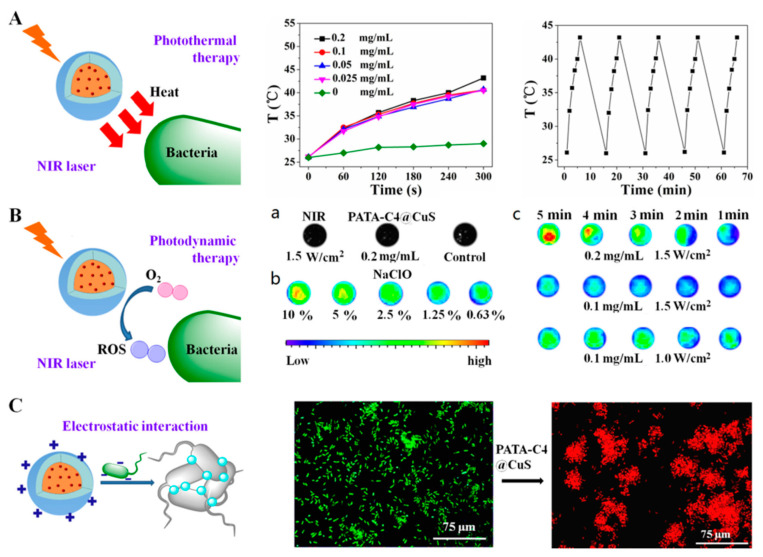
(**A**): Photothermal temperature change of PATA3-C4@CuS at different concentrations under NIR laser irradiation. (**B**) Fluorescence imaging of ROS generation detected by DCFH-DA assay: (**a**) single PBS and PBS containing PATA3-C4@CuS/NIR treated with DCFH-DA (black map indicates no ROS generation), (**b**) NaClO solution at different concentrations treated with DCFH-DA, and (**c**) solution containing PATA3-C4@CuS treated with DCFH-DA under various conditions. (**C**) Fluorescence micrographs of *B. amyloliquefaciens* stained with acridine orange (AO) and ethidium bromide (EB) before and after treatment with PATA3-C4@CuS. Reproduced with permission from Ref. [9]. Copyright 2017 American Chemical Society.

CuS nanomaterials were also utilized with different polymers to achieve synergistic photoactivated antibacterial activity. Dai et al. [9] synthesized a collection of poly(5-(2-ethyl acrylate)-4-methylthiazole-g-butyl)/copper sulfide nanoclusters (PATAC4@CuS), which were applied to levofloxacin-resistant Gram-negative and Gram-positive bacteria for efficient capture and effective ablation by NIR laser irradiation. The nanoclusters not only showed excellent photothermal activity under NIR irradiation, but also demonstrated admirable photodynamic properties (Figure 4). PATAC4@CuS nanoclusters showed excellent antibacterial activity against *B. amyloliquefaciens*, *E. coli, P. aeruginosa*, and levofloxacin-resistant *S. aureus* at 5.5 μg·mL^−1^ under NIR laser irradiation (980 nm, 1.5 W·cm^−2^, 5 min). The heat and ROS generated from the NIR-irradiated PATAC4@CuS nanoclusters were stated as the reasons for the effective elimination and prevention of regrowth of the bacteria. The nanocluster also showed wound healing potential in bacteria-infected rat wounds without nonspecific harm to normal tissue. In another similar experiment, Wang et al. [75] introduced a thiol-terminated, alkyl-containing short-chain poly(ethylene glycol) (HS-(CH_2_)_11_-(OCH_2_CH_2_)_6_-OH, abbreviated as MUH, onto CuS nanoclusters (NCs) to achieve photothermal antibacterial activity under NIR irradiation. The MUH-coated CuS NCs exhibited excellent stability in solutions with various pH values and stayed stable in pure water for at least 10 months. The MUH-coated CuS NCs showed strong antibacterial activity toward *E. coli* bacteria at 800 μM (76.8 μg·mL^−1^) concentration.

### 4.4. CuS with Protein

Despite numerous advantages of CuS nanomaterials for biomedical applications, toxicity remains an issue that needs to be resolved. To address this issue, researchers have tried to coat CuS with proteins, especially bovine serum albumin (BSA). BSA is selected as a coating for CuS due its various advantageous properties, such as versatility, non-toxicity, stability, and biodegradability. Additionally, its functional groups allow easy binding and capping of nanomaterials, such as CuS.

**Figure 5:**
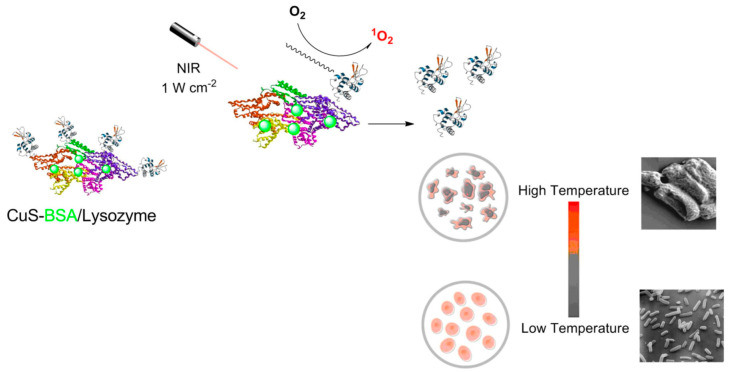
Schematic illustration of PDT and PTT mediated antibacterial activity of BSA-CuS nanocomposite. Reproduced with permission from Ref. [77].

Zhao et al. [76] applied BSA-CuS NPs to the treatment of diabetic wound infection in vivo as a method of photothermal therapy. A facile biomineralization method was used to synthesize BSA-CuS nanomaterials, which were successfully used to destroy *A. baumannii*, *S. aureus*, and *S. haemolyticus* under NIR irradiation (808 nm, 8.0 W·cm^−2^) for 10 min at 200 mg·L^−1^ concentration. Both in vitro and in vivo experiments were performed in this case. The BSA-CuS NPs showed excellent NIR-mediated photothermal antibacterial activity, with low cytotoxicity, in vivo toxicity, and excellent water solubility. Dual-mode PTT and PDT was applied using Ce6-labeled BSA-CuS NPs, enabling a synergistic effect against *E. coli and S. aureus* both in vitro and in vivo [10]. Most importantly, the same NIR light source was utilized to generate the ROS required for PDT activity and for PTT. The CuS NPs were prepared in the presence of BSA, which prevents aggregation of the CuS NPs. BSA also acts as a linker between the CuS and Ce6 molecules through amide bonds. The Ce6-labeled BSA-CuS NPs showed an overall bacterial killing efficacy of 97% in vitro due to the synergistic effects of combined PTT and PDT. The nanocomposite also successfully eliminated bacterial infection in vivo. BSA was also utilized as a template to synthesize uniform CuS NPs by a biomineralization method, and its NIR-mediated photothermal antibacterial activity against *E. coli* and *S. aureus* was evaluated (Figure 5). The as-prepared BSA-CuS nanocomposites showed good biocompatibility with skin fibroblast cells. Moreover, the nanocomposites showed excellent antibacterial activity under NIR light (980 nm, 1.59 W·cm^−2^). The biocompatible CuS-BSA/lysozyme nanocomposite was also effective against *B. subtilis* and *E. coli* under NIR irradiation (980 nm, 0.7 W·cm^−2^) [77].

### 4.5. CuS with Metals

The use of intrinsic antibacterial agents, such as Ag [83,86], Au [36], and ZnO [20,86], in combination with CuS to achieve photothermal antibacterial activity, is still in the infancy stage. Addae et al. [78] demonstrated the strong photothermal antibacterial potential of Au/CuS NPs against *Bacillus anthracis*. The antibacterial effect was dependent on both the NP concentration and treatment time. Similarly, Park et al. [89] showed the photothermal antibacterial potential of Au@CuS against *E. coli*. The Au@CuS yolk-shell nanomaterials on a PDMS film induced excellent photothermal sterilization against *E. coli*, where the photothermal efficiency of the nanocomposite reached 79.8%. Further research in this area is still necessary to completely understand the antibacterial mechanism of the nanocomposites. 

## 5. Conclusions and Future Perspectives

In conclusion, it is certain that research to tackle MDR bacteria and their associated infections has seen remarkable advancements in recent years. The urgent need for alternatives to current antibiotics is well recognized. Consequently, therapeutic agents for PDT and PTT are prospective game-changers for antibacterial therapy. CuS is an especially promising material due to its multiple beneficial properties as an antibacterial agent. As discussed earlier, CuS absorbs NIR light through an LSPR, which originates from the collective oscillation of holes rather than electrons in other widely used photoactive nanomaterials such as Ag and Au. Furthermore, Cu^2+^ is recognized as an important trace element in the human body, stimulating the wound healing process. Therefore, the advantages of CuS over other photoactive nanomaterials are noteworthy. The most important aspect is that the PDT and PTT can be effectively utilized with CuS nanomaterials to achieve excellent antibacterial activity. Furthermore, the odds of bacterial resistance are almost non-existent. The multimodal strategy of combining PDT and PTT is also possible with CuS. Despite these remarkable antibacterial properties and advantages, some challenges still remain to be resolved. First and foremost, all the reported studies on PDT and PTT with CuS were performed under laboratory conditions. Hence, the same significant antibacterial activity remains elusive in clinical trials. There is also the ever-present issue of the toxicity of nanomaterials, such as CuS, which requires attention. The biocompatibility, target specificity, and stability of CuS can be amended with the help of functional nanoconjugates. Nevertheless, the synthesis of CuS nanomaterials is somewhat complicated in comparison with that of other well-known antibacterial agents, along with the relatively high cost of the former. This pricey aspect of CuS synthesis could hinder its application in large-scale clinical settings. Hence, the affordable preparation of CuS by economical and green methods is a future direction for large-scale synthesis. The high dose of CuS nanomaterials required to achieve antibacterial activity also needs to be addressed. Different synthesis methods and various CuS morphologies can prospectively be utilized to diminish the problem of high dosage. In summary, we expect the emergence of more therapeutic strategies involving CuS nanomaterials or nanocomposites to combat bacterial infection in clinical applications in the global research arena.

## Figures and Tables

**Table 1:** Antibacterial activity of phoactivated CuS nanomaterials.

Photoactivated CuS	Antibacterial Mechanism	Light Source	Bacteria	Efficacy	Ref.
CuS NDs	PTT & Cu^2+^ release	NIR (808 nm, 2.5 W·cm^−2^)	ESBL *E. coli*, MRSA	~100%	[46]
CuS NPs	PTT	UV (400 mW·cm^−2^)	*E. coli*, *S. aureus*	99.99%	[72]
CuS NPs	PTT	NIR (800, 900, and 1000 nm, 0.26 W·cm^−2^)	*E. coli*, *S. aureus*	95% (>5 h)	[43]
CuS MSs, NSs, NPs	PDT, PTT	Simulated solar light and NIR (808 nm, 1.5 W·cm^−2^)	*E. coli*	Effective	[7]
CuS/GO	PTT	Visible light (0.2 W·cm^−2^)	*E. coli*, *S. aureus*	99.98%	[73]
PEG-CuS@g-C_3_N_4_	PTT	NIR (808 nm, 2.5 W)	*E. coli*, *S. aureus*	~99%	[74]
Ti_3_C_2_T_x_@CuS	PDT, PTT	NIR (808 nm, 1.5 W)	*E. coli*, *S. aureus*	~99%	[8]
CuS-BP	PDT, PTT	NIR (808 nm, 2.5 W)	*P. aeruginosa*, *S. aureus*	~100%	[47]
PATAC4@CuS	PDT, PTT	NIR (980 nm, 1.5 W·cm^−2^)	*Bacillus amyloliquefaciens*, *E. coli*, *P. aeruginosa,* Levofloxacin-resistant *S. aureus*	Effective	[9]
MUH@CuS NCs	PTT	NIR	*E. coli*	~100%	[75]
BSA-CuS NPs	PTT	NIR (808, 8.0 W·cm^−2^)	*A. baumannii*, *S. aureus*, *S. haemolyticus*	Effective	[76]
Ce6-labeled BSA-CuS NPs	PDT, PTT	NIR (980 nm, 1.59 W·cm^−2^)	*E. coli*, *S. aureus*	97%	[10]
CuS-BSA/lysozyme	PTT	NIR (980 nm, 0.7 W·cm^−2^)	*B. subtilis*, *E. coli*	~100%	[77]
Au@CuS	PTT	NIR	*E. coli*	~100%	[78]

Abbreviations: Near-infrared, NIR; Methicillin-resistant *Staphylococcus aureus*, MRSA; Extended-β-lactamase, ESBL; Polyethylene glycol, PEG; Graphene oxide, GO; Microsphere, MS; Nanosheet, NS; Nanoparticle, NP; Nanodot, ND; poly(5-(2-ethyl acrylate)-4-methylthiazole-g-butyl)/copper sulfide nanoclusters, PATAC4@CuS; thiol-terminated, alkyl-containing short-chain poly(ethylene glycol) (HS-(CH_2_)_11_-(OCH_2_CH_2_)_6_-OH, MUH.

## Data Availability

Not applicable.
